# Remembering conversation in group settings

**DOI:** 10.3758/s13421-024-01630-8

**Published:** 2024-09-05

**Authors:** Sarah Brown-Schmidt, Christopher Brett Jaeger, Kaitlin Lord, Aaron S. Benjamin

**Affiliations:** 1 Department of Psychology and Human Development, Vanderbilt University, 230 Appleton Place, Nashville, TN 37203 USA; 2 School of Law, Baylor University, Waco, TX USA; 3 Department of Psychology, University of Illinois Urbana-Champaign, Urbana-Champaign, IL USA

**Keywords:** Conversation, Recall, Memory, Conversational role

## Abstract

Individuals can take on various roles in conversation. Some roles are more active, with the participant responsible for guiding that conversation in pursuit of the group’s goals. Other roles are more passive, like when one is an overhearer. Classic accounts posit that overhearers do not form conversational common ground because they do not actively participate in the communication process. Indeed, empirical findings demonstrate that overhearers do not comprehend conversation as well as active participants. Little is known, however, about long-term memory for conversations in overhearers. Overhearers play an important role in legal settings and dispute resolution, and it is critical to understand how their memory differs in quality and content from active participants in conversation. Here we examine – for the first time – the impact of one’s conversational role as a speaker, addressee, or overhearer on subsequent memory for conversation. Data from 60 participants recalling 60 conversations reveal that after a brief delay, overhearers recall significantly less content from conversation compared to both speakers and addressees, and that the content they do recall is less accurately sourced to its actual contributor. Mnemonic similarity is higher between active conversational participants than between active participants and overhearers. These findings provide key support for the hypothesis that the process of forming common ground in interactive conversation shapes and supports memory for that conversation.

**Supplementary Information:**

The online version contains supplementary material available at 10.3758/s13421-024-01630-8.

## Introduction

Group conversations are complex and dynamic, with individuals shifting and switching roles as topics change and the discussion evolves. There are moments in which an individual may guide the conversation toward a resolution or away from a dispreferred topic, and other moments in which that individual may passively witness exchanges between others in the group. There are also individual differences in how one contributes to conversation (e.g., Leaper & Smith, 2004; Tenenbaum et al., 2011). Yet little is known about the consequences of serving in these varied roles for how one remembers conversation. Memory for conversation plays an important role in ongoing cognitive processes and in dispute resolution (e.g., Brown-Schmidt et al., 2023); as such, studies of long-term memory are an important part of understanding how conversations influence human behavior in high-stakes settings.

Empirical studies of conversational memory have attempted to quantify the completeness and accuracy of conversational memory, and explain how memory for conversation relates to factors such as semantic characteristics of what was said, but these studies have focused on memory of active conversational participants (Keenan et al., 1977; MacWhinney et al., 1982; Pezdek & Prull, 1993). Yet communicative settings don’t always involve active conversation – in many cases, a person may be an overhearer or a marginal participant while occupied by another task (Kuhlen & Brennan, 2010; Brennan et al., 2010). Here we examine the effect of one’s role in conversation – active participant or nearby overhearer – on later memory for that conversation.

Classic views on the mechanisms of conversation posit that as conversational partners interact with each other, they develop representations of what information is mutually known – knowledge that the partners know that each other knows (Clark & Marshall, 1978). This mutual knowledge, or *common ground* is thought to grow as one partner provides information (e.g., *I’m from Seattle*), and the other partner acknowledges that it was understood (e.g., *Oh wow, I’ve never been*). Empirical studies of conversation commonly point to the finding that as partners discuss new information in conversation (e.g., *You know that new Korean restaurant that opened up on Green Street*), they develop short-hand ways of referring to it (e.g., *that Korean restaurant*). This abbreviation of reference is thought to reflect the development of common ground through conversation (Krauss & Weinheimer, 1966; Wilkes-Gibbs & Clark, 1992; *inter alia*). However, if one of these partners then speaks to a new partner who was not actively participating in that first conversation, they use longer and more descriptive referential terms to accommodate the new partner’s lack of knowledge (Wilkes-Gibbs & Clark, 1992). Speakers similarly use longer referential expressions when speaking to a group of people, only some of whom are familiar with the terms, compared to a group of people, all of whom are familiar with them (Yoon & Brown-Schmidt, 2019). Moreover, other findings show that conversational participants exhibit coordination both in the words they use (Brennan & Clark, 1996; Manson et al., 2013) and in their visual attention to things in the world that they discuss (Richardson et al., 2007, 2008), further reflecting the shared representations that emerge through conversation.

Critically, because active conversational partners design what they say in a way that is intended for active partners to understand – but not necessarily for an overhearer to understand – the overhearer may need to use more inferential processes to understand what is being said (Clark & Schaefer, 1992), or may be entirely unable to reach a complete understanding of the content. Indeed, when an overhearer tries to follow along with a task-based conversation by listening in on a pair of active participants, they are less accurate at the task (Schober & Clark, 1989), indicating that simply hearing what others are saying does not support understanding in the same way as being actively engaged in the conversation. Findings like these indicate that overhearers are less successful at interpreting conversation in the moment, which suggests they may also have poorer memory for conversation than active participants. On the other hand, being an active participant in conversation is a cognitively demanding activity that requires listening to one’s partner while simultaneously planning what to say next (Bögels et al., 2015), and monitoring one’s partner for their understanding while speaking (Clark & Krych, 2004). When alleviated of the communicative pressure to tailor one’s own behavior to that of the conversational partner, this may create an opportunity for non-active participants to develop a balanced representation of the contributions of the conversational participants.

There are very practical reasons for why it is important to understand the downstream consequences are of being an overhearer on the ability to subsequently remember what was said in an overheard conversation. Consider the following transcribed statement that was made by a person (CJ) being interviewed by a Sherriff (SS) of the Floyd County Police Department^1^:

SS: *Alright just tell me in your own words and (inaudible* –* moving microphone) be uh, what you heard uh, concerning this death of Isaac Dawkins.*

CJ: *I overheard Joey Watkins talking to a crowd of people [sic] Home Depot parking lot about how they watched the boy get into his car and leave and follow him out into the highway and whoever was with him was supposedly Booney* – *and Joey heard him telling people that he didn't know that he was really gone do it and then he said next thing he knew when they pulled up beside him or whatever that boom* –* I mean he shot him.*

At trial,^2^ this same individual responds to questions (Q) with the following answers (A):

Q: How come you are at the Home Depot parking lot at night?

A: That is where everybody was hanging out on the weekends.

[…]

Q: And you heard Joey Watkins say something?

A: Yes, ma’am.

Q: And he wasn’t talking to you though?

A: No.

Q: No? What did you hear him telling the people – the person or people around him?

A: He was just bragging about shooting Isaac.

[…]

Q: What did he say?

A: *He said that they watched Isaac leave the college and they sped up behind his car and shot and then just shot again, and he just kept going on about his business.*

What can the science of memory for conversation tell us about the quality of memory for conversation in cases where the reported memory comes not from a conversation that a person was actively involved in, but instead, a conversation that was overheard? Although this example illustrates an extreme case of the real-world significance of an alleged memory for overheard conversation,^3^ conversational memory, including memory for overheard conversations, is a common and critically important type of memory in legal settings and other governmental proceedings (Davis & Friedman, 2007; Hope, et al., 2019; Neisser, 1981; Pezdek & Prull, 1993). As we shall see, the existing literature on conversational memory provides some clues about how one’s role in a conversation might affect memory for the content of that conversation.

### Memory for conversation

Following a real-world conversation, there is limited opportunity to objectively evaluate the completeness and accuracy of a memory report if there is no audio or video record of that interaction (cf., Neisser, 1981). In contrast, in experimental studies of conversation, the researcher can invite participants to the lab to engage in a conversation, record that conversation, and then later ask participants to recall that conversation in detail. The fidelity of memory can then be objectively assessed by comparing the recording of the conversation to the memory report. In the present research, we employ a recall procedure to probe memory in which participants are asked to recall (in writing) as much of a prior conversation as possible. This type of recall procedure is similar to a real-world situation where a person attempts to recall what was said in a prior conversation after some delay. In business and certain diplomatic and political settings, documentation of a conversation using a “memcon” (memoranda of conversation) provides a record of what was said, and may be completed by one of the conversational participants or by a team of assistants listening in on the conversation (Brown-Schmidt et al., 2023). Example memcons can be found at the Clinton^4^ presidential library as well as at the National Security Archive.^5^

A key finding in the literature on memory for conversation is that memory for what a person said in conversation themselves is often superior to memory for what was said to them. This finding likely reflects the influence of known memory phenomena that information that is generated or produced tends to be better remembered after a delay compared to information that is read or heard (MacLeod et al., 2010; Slamecka & Graf, 1978; Zormpa et al., 2019a, 2019b). For example, Ross and Sicoly (1979) report that participants were able to recall significantly more of their own contributions than their partner’s contributions to a conversation after a 3- to 4-day delay (5.6% vs. 2.6%). Likewise, Miller et al. (1996) report that after a 15- to 20-min discussion of liberal arts course requirements, participants recalled significantly more of their own contributions than their partners (the data are not reported in a way that allows calculation of % recall). Similarly, Brown-Schmidt et al. (2023) report that after a 1-week delay, participants recalled significantly more of their own contributions to the conversation compared to their partner’s (16.7% vs. 12.7%).

Some studies do not replicate the generation benefit in conversational memory, including Benoit and Benoit (1988), who report 15% free recall of one’s own statements versus 17% free recall of the partner’s statements after a 7-min delay (also see Stafford & Daly, 1984; Stafford et al., 1987). One explanation offered by Miller et al. (1996) for the inconsistency in findings across studies relates to the perceived importance of the conversational topic (e.g., whether it’s a routine get-to-know-you conversation where some of the content of what one said themselves might be seen as too trivial to report, compared to a more substantive conversation). Other findings using recognition memory procedures find a clear generation benefit to memory for images that were described by one’s self versus by one’s partner in conversation (McKinley et al., 2017; Yoon et al., 2016, 2021), and for the image labels (Nault et al., 2023) and phrases said versus heard in conversation (Fischer et al., 2015; also see Jurica & Shimamura, 1999). Naming pictures (compared to repeating or reading the name) promotes later memory for both the picture (Zormpa et al., 2019a, 2019b) and for the name itself (Zormpa et al., 2019a, 2019b), further illustrating how producing and generating linguistic information supports memory for that experience more generally.

We are unaware of any research directly comparing conversational recall for active conversational participants and overhearers, but research indicates that active participation confers benefits over simply hearing a *recording* of a conversation. For example, MacWhinney et al. (1982) report that recognition memory for conversational utterances was better for active participants compared to strangers who watched a video of the conversation. Similarly, Benoit and Benoit (1994) report greater cued recall and recognition memory for conversational participants compared to “observers” who listened to recordings of prior conversations.

This advantage to conversational memory for active engagement compared to watching a video may relate to the way in which conversational partners engage with each other and form common ground. For example, research measuring the use of common ground in online language processing reveals the successful use of common ground representations in interactive conversation, but not when new participants listened to recordings of those conversations (Brown-Schmidt, 2009). Further, evidence that the process of interactively engaging in conversation shapes *memory* comes from McKinley et al. (2017). In that study, participants formed common ground as they described images multiple times; when common ground for a given image description was more firmly established, subsequent recognition memory for those images was superior. Similarly, Nault et al. (2023) report that the degree of common ground formed for image labels in a referential communication task was positively associated with verbatim recall of those image labels after a 1- to 2-week delay.

### The present research

The present research serves as the first test in the experimental literature of the hypothesis that active participation in conversation confers benefits to memory for the contents of conversation compared to a case where one overhears that conversation. As a starting point for this research question, we intentionally examine a situation in which the overhearer is co-present but engaged in a concurrent task (here, a computerized chess game). We selected this scenario because overhearing often involves dual-task processes. After all, if you’re sitting in the same room as the conversational participants, but not talking to them, it’s likely because you’re engaged in some other task (e.g., doing a puzzle, eating, or maybe even trying to appear as if you’re *not* eavesdropping).

When interacting with a distracted listener, speakers say less and change the content of what they say (Dickinson & Givon, 1995; Pasupathi & Hoyt, 2009, 2010), perhaps in recognition of the challenges that distraction poses for the listener. But an overhearer does not have a privileged role in conversation and the speaker will not shape the content to match the challenges they face. In addition, the overhearer will not have access to the benefits to understanding and memory that arise from monitoring eye gaze and other interpersonal cues (Argyle & Cook, 1976; Pelachaud et al., 2005). So, there are good reasons to anticipate that dual-tasking demands will impair memory for the conversation for overhearers. But it is not clear which aspects of conversational memory will be impaired. Memory impairments, like the type owing to distraction, provide an assay of which aspects of remembering are more resistant to disruption (Benjamin, 2010). This study serves as a first foray into the real costs of overhearing an ordinary conversation while doing an ordinary task, and can reveal a profile of which aspects of conversation are more fragile, and which are more robust, to distractions associated with overhearing.

We anticipate that overhearers will exhibit poor memory for conversation due to disruption and the lack of common ground, but another perspective on the issue is possible. Participating in conversation is a cognitively demanding activity, and those demands may interfere with committing the content of the conversation to memory. Speakers must shift attention to upcoming to-be-uttered information at just the right time in order to plan the words in sequence and at the right time (Bock, 1982; Griffin & Bock, 2000; MacDonald, 2016; Nozari & Dell, 2012). In conversation, turn-taking is rapid with few pauses (Sacks et al., 1974; Stivers et al., 2009), meaning that conversational participants begin preparing their own speech while they are listening to what their partner is saying (Bögels et al., 2015). Absent these demands, the overhearer may be in the privileged position of being able to process all of the speech without the concurrent pressure of monitoring the addressee for understanding (Clark & Krych, 2004) and planning their own upcoming utterances. If so, an overhearer advantage might be observed, particularly when comparing active participants’ memory for utterances for which they were the addressee (and thus not benefitting from a generation-boost to memory), to overhearer’s memory for the very same utterances.

### Method

The data for this study were collected in the first author’s laboratory at Vanderbilt University between 2017 and 2018, with data coding and analysis through 2023. The study was pre-registered (https://osf.io/pjv2c) after the data from three groups had been recorded but prior to inspection or analysis of any data. This study was approved by the Vanderbilt University Institutional Review Board, and all participants provided informed consent prior to participation.

#### Participants

Participants were recruited in groups of three from the Vanderbilt University research participation pool and were compensated with partial course credit or $20 for a 2-h study time-slot. Participation was restricted to individuals between 18 and 25 years of age who self-reported being a native speaker of North American English and who reported not having problems with hearing or speech. A total of 21 groups of three participants completed the study. The data from one group was excluded from analysis because two participant recalls were lost due to computer error. The final dataset submitted to analysis included 20 groups of three participants, as pre-registered. This sample size was determined a priori and was selected because it was the largest sample size that could be feasibly collected.

#### Procedure

The three participants were seated in a room in the first author’s laboratory approximately 4 ft apart in a triangular arrangement. Two participants were seated at one table, and the third participant was seated at a nearby table. An audio recording device was set up on the first table to record the entire interaction (video was not recorded). After signing consent forms, individuals were randomly assigned to the roles of participants A, B, and C. An experimenter instructed the participants that to begin, participants A and B would sit at the first table together and have a conversation for 5 min while participant C waited at the second table. A laptop at the second table was set up for participant C to play a computerized chess game while they waited. Most participants reported limited chess ability and no participant beat the computer, which was placed on an easy setting. Critically, participant C was seated the same distance away from A and B as A and B were from each other, and thus had the opportunity to overhear the entirety of their conversation. We gave participant C the task of playing chess so that they would have something to do during the conversation, decreasing the chances that they would interrupt or be invited to join in on the conversation. The task of chess was selected to be familiar enough to most participants that they could attempt to play, yet not require rapid responses to visual or auditory stimuli (such as a typical shooting or driving type video game) or processing of verbal information that might interfere with comprehension of the conversation.

The participants were instructed that they would have three total conversations, rotating partners each time so that each person would have a chance to talk to each of the other two people. The participants’ instructions were as follows: “The two of you [A & B] will be seated here and we are going to record your voices while you have a short conversation. You will need to wear microphones so we have a good recording of your voice. Meanwhile, C, you are going to sit over here and play this game for points.” For each conversation, a piece of paper with a conversation-starter prompt was provided on the table, though participants were free to talk about anything they wished. The first conversation was between participants A and B and the conversational starter was favorite music; the second conversation was between participants A and C and the topic was favorite TV shows; the third conversation was between participants B and C and the topic was living in Nashville. Between conversations, the participants switched seats so that the conversing participants sat together at the first table and the non-conversing participant sat at the second table. In cases where the non-conversing participant interrupted, they were reminded not to interrupt by a research assistant sitting in the room (interruptions were uncommon). Prior to the conversation, participants were not told there would be a memory test.

Following the three conversations, the participants watched a video about astrophysics for 20 min. This was intended as a filled delay task, designed to mimic a real-world situation where a person has a conversation and then attempts to recall it after a delay. Next, participants were brought to three separate (new) locations in the laboratory and each was seated at a computer for a (surprise) free recall test of the three conversations. The participants were instructed that they would be asked to recall in writing as much as they could from each conversation. They were asked to write out the conversation word-for-word using as much detail as possible, including who said what. The participants were given 10 min to recall each conversation and were given separate recall sheets with the instruction to recall the “first,” “second,” or “third” conversation, in that order. The entire experiment lasted less than 2 h. See Table 1 for the timeline of the procedure.

**Table 1:** Timeline of study procedure

Time-keeping	Task	Person A	Person B	Person C
10 min	Consent/Instructions			
5 min	Conversation 1: Music	*Converse with B*	*Converse with A*	*Play chess/overhear*
5 min	Conversation 2: TV show	*Converse with C*	*Play chess/overhear*	*Converse with A*
5 min	Conversation 3: Living in Nashville	*Play chess/overhear*	*Converse with C*	*Converse with B*
20 min	Watch video (filled delay)	*Watch video*	*Watch video*	*Watch video*
10 min	Recall 1	*Recall Conversation 1*	*Recall Conversation 1*	*Recall Conversation 1*
10 min	Recall 2	*Recall Conversation 2*	*Recall Conversation 2*	*Recall Conversation 2*
10 min	Recall 3	*Recall Conversation 3*	*Recall Conversation 3*	*Recall Conversation 3*

#### Data coding

In total, this experiment generated 60 5-min conversations. Each conversation was transcribed word-for-word, including who said what (participant A, B, or C). Following standard coding conventions in conversational memory research, the utterances were broken into “idea units,” which are typically defined as "the smallest unit of meaning that has informational or affective value; it represents the gist of each thought expressed by the interactants" (Stafford et al., 1989, p. 600; also see Ross & Sicoly, 1979; Stafford & Daly, 1984; Stafford et al., 1987). The conversational idea units were numbered and the speaker was identified.

In addition to the conversations, this experiment generated 180 conversational recalls. The recalls were broken into idea units using the same coding procedure as the conversation. Note that for each of the 60 conversations, two of the recalls were from the conversational participants, and the third recall was from the participant who overheard the conversation. Note that one participant recalled one conversation twice and skipped the recall of the third conversation; the second recall of the second conversation was not analyzed. The final analysis is thus based on 179 conversational recalls. An example excerpt from a de-identified conversation, along with the recalls by the two active participants, is provided in Table 2.

**Table 2:** Example conversation between participants B and C and their individual recalls

Speaker	Transcript	B's recall	C's recall
C	And definitely wanna like check out a…	He said how we was interested in seeing	One of the things I want to do here is see some
C	Predators game	Predators games	Predators games
C	at some point	-	-
B	Yeah, my um…floor went to a	and I remarked how my floor went to	Yeah, a few of my friends went to a
B	Predators pre-season game	a preseason game	Predators preseason game
B	a couple weeks ago	a couple weeks ago	a couple weeks ago
C	Oh damn	-	Oh that’s dope
B	So that was pretty cool	-	-
C	Yeah	-	-
B	Um…are you a	I asked if he was a	Yeah. So are you a
B	Bruins fan too or	Bruins fan as well	Bruins fan?
C	No, I’m actually a	and to my horror he replied that he was a	No haha, I’m actually a
C	Canadiens fan [laugh]	Canadiens fan	Canadiens fan

The de-identified transcript is broken into idea units uttered by participants B and C, and matched to the recalls. Participant A was in the role of overhearer and did not accurately recall any details of this conversational excerpt. Dash marks (-) indicate an idea unit that was not recalled

Each conversation was then compared to the three recalls to quantify the completeness and accuracy of recall. We used a gist recall procedure where a conversational idea unit was coded as recalled if the person recalled the gist meaning of the conversational idea unit, regardless of whether the surface linguistic form was reproduced. For example, in the excerpt in Table 2, the idea unit “*Oh damn*” was coded as recalled when participant C recalled it as “*Oh that’s dope,*” as this recall reproduced the general meaning of this idea.

We report three primary analyses of conversational memory: *completeness* of recall, *accuracy* of recall (and source accuracy), and *similarity* of recall across group members. Completeness quantifies the proportion of information present in the original conversation that was recalled by participants after the delay. Accuracy quantifies whether each recall idea unit reflects an accurate recall of something said in the conversation, an inaccurate recall (i.e., something not said in the original conversation), or other extrapolations or commentary. The extrapolations and commentary typically included reports of internal thoughts or inferences based on the conversation. For example, one participant accurately recalled that her partner mentioned a local restaurant but then wrote “*I immediately jumped at that”* (coded as a commentary), “*saying that although I’ve never been there before”* (coded as accurate recall), *“I’ve heard a lot about it*” (coded as inaccurate recall, as this idea was not expressed in the conversation). Source accuracy further quantifies whether or not the recall idea unit correctly identifies the source of each idea unit or not (e.g., whether participant A, B, or C uttered it), excluding all recall of idea units in which the source is not specified. Finally, we compare the recalls of the three participants within a triad to understand the similarity of recall across persons.

To calculate intercoder reliability, two groups were randomly selected to be coded independently by a second coder. Of all idea units produced in the two conversations, the coders were in agreement as to whether or not a given idea unit was recalled or not 94.2% of the time; this corresponds to a Kappa of 0.834 (SE = 0.01), indicating almost perfect agreement (calculated using GraphPad QuickCalcs (2023).^6^ Of all recall idea units, the coders were in agreement as to whether or not a given recall idea unit reflected an accurate recall of something said in the conversation 90.2% of the time, corresponding to a Kappa of 0.545 (SD = 0.04, indicating moderate agreement). Most disagreements concerned whether a recall idea unit reflected an extrapolation from the conversation or an accurate recall. After independently coding these two groups, the second coder then reviewed the coding for all groups and made final coding decisions; this dataset was used as the final dataset for analysis.

### Results

Following the pre-registration (https://osf.io/pjv2c), our inferential statistics include mixed effects logistic regression analyses of recall completeness and source accuracy as a function of participant role (speaker, listener, or overhearer). In the period of time between completing the pre-registration and preparation of this article, a number of new findings in the laboratory, as well as our discovery (and re-discovery) of existing papers in the literature on conversational memory additionally led us to examine the accuracy of recall and the similarity of recall among the conversational participants. Although the analyses of recall accuracy and recall similarity were not pre-registered, these analyses were planned prior to any examination or analysis of the data, with the exception of one post hoc analysis of similarity which was planned after inspection of the data. We first present descriptive statistics from the dataset and then present the inferential statistics.

#### Descriptive statistics

The 60 conversations contained an average of approximately 1,001 words (SD = 136) and 221 idea units (SD = 35), with each idea unit on average containing 4.58 words (SD = 0.48). The 179 recalls in this analysis each contained approximately 288.18 words (SD = 122.64) and 62.09 idea units (SD = 25.99), with each recall idea unit on average containing 4.71 words (SD = 0.88). Of the total 11,114 idea units produced during the recall phase, 81.09% reflected an accurate recall of something that was said in the conversation (9,012 idea units), 14.77% reflected extrapolations or other commentary (1641 idea units), and only 4.15% reflected an inaccurate recall (461). Focusing on those accurate recalls, of the 13,239 conversational idea units included in the 60 conversations, speakers accurately recalled 29.83%; addressees recalled 22.86%, and overhearers recalled 12.05%. This pattern of limited yet generally accurate recall is a common finding in similar studies of conversational recall after brief delays (Brown-Schmidt et al., 2023; Diachek & Brown-Schmidt, 2022).

#### Completeness of recall

**Fig. 1:**
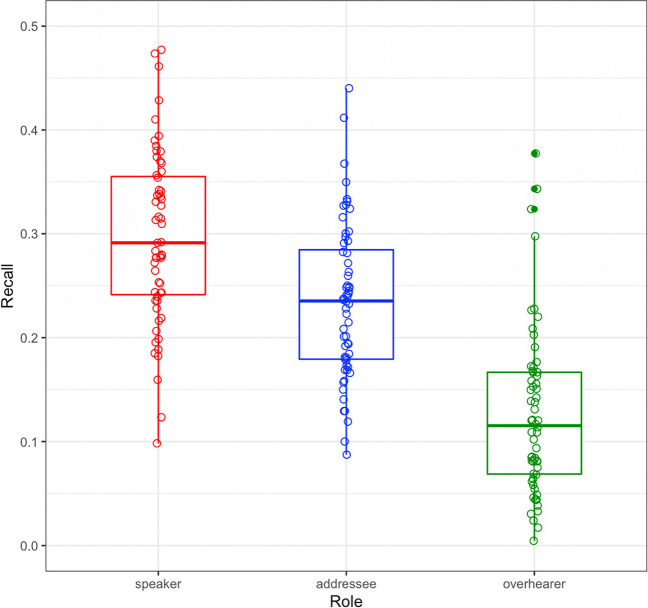
Recall completeness for the 13,239 idea units across 60 conversations as a function of participant role (speaker, addressee, overhearer). For illustrative purposes, individual participant means are indicated by circles

The analysis of recall completeness examined whether or not each of the total 13,239 conversational idea units was later recalled by the person who produced that utterance (the speaker), the person to whom it was addressed (the listener), and by the overhearer who was positioned to hear everything said in the conversation, but who was not an active conversational participant (Fig. 1; this and the following figures were plotted with ggplot2; Wickham, 2016).

The completeness data were analyzed using mixed-effects logistic regression using the glmer function in lme4 (Bates et al., 2015) in R version 4.1.2 (R Core Team, 2021). The fixed effects in the model included conversational role (speaker, addressee, overhearer) as specified in the pre-registration; the role was coded using Helmert contrasts with the first contrast comparing active conversational participants (speaker, addressee) to the overhearer, and the second contrast directly comparing speaker and addressee. In addition, we included two control variables that were not specified in the pre-registration, but that we hypothesized might be important predictors of idea unit memorability based on recent research (Brown-Schmidt et al., 2023; Lord & Brown-Schmidt, 2022). These factors were *idea unit order* in the conversation (centered and scaled by /100), and *idea unit length* in terms of the number of words (centered and scaled by /10). To identify a parsimonious random effects structure for the model (Matuschek et al., 2017), we used the buildmer function (Voeten, 2020). The input to the buildmer function included all fixed effects as well as random effects of individual participant, conversational group, and individual idea unit, and all possible random slopes for the fixed effects. The output of the buildmer process (Table 3) included random intercepts by participant and idea unit, and random slopes by participant for Role and IU order. Random effects by group were removed from the model during this process, suggesting that clustering of responses by group was not substantial.

**Table 3:** Recall completeness: results of mixed-effects logistic regression analysis for 39,717 binary data points (13,239 idea units (IUs) recalled, or not, by each of three individuals) and 60 participants, as a function of participant role (speaker, addressee, overhearer), IU order in the conversation, and IU length (in words)

Fixed effects	Estimate	SE	z-value	*p*-value	OR
(Intercept)	-1.764	0.066	-26.743	< .0001	0.171
IU order	-1.082	0.088	-12.328	< .0001	0.339
Role1 (S = .25, A = .25, O = -0.5)	1.685	0.151	11.133	< .0001	5.393
Role2 (S = .5, A = -.5, O = 0)	0.442	0.052	8.549	< .0001	1.555
Idea Unit length	0.299	0.064	4.682	< .0001	1.348
Random effects	Variance	SD	Corr		
IU (intercept)	0.755	0.869			
Participant (intercept)	0.235	0.485			
IU order	0.415	0.644	0.320		
Role1	1.241	1.114	-0.330	-0.170	
Role2	0.099	0.315	0.050	-0.140	0.080

**Fig. 2:**
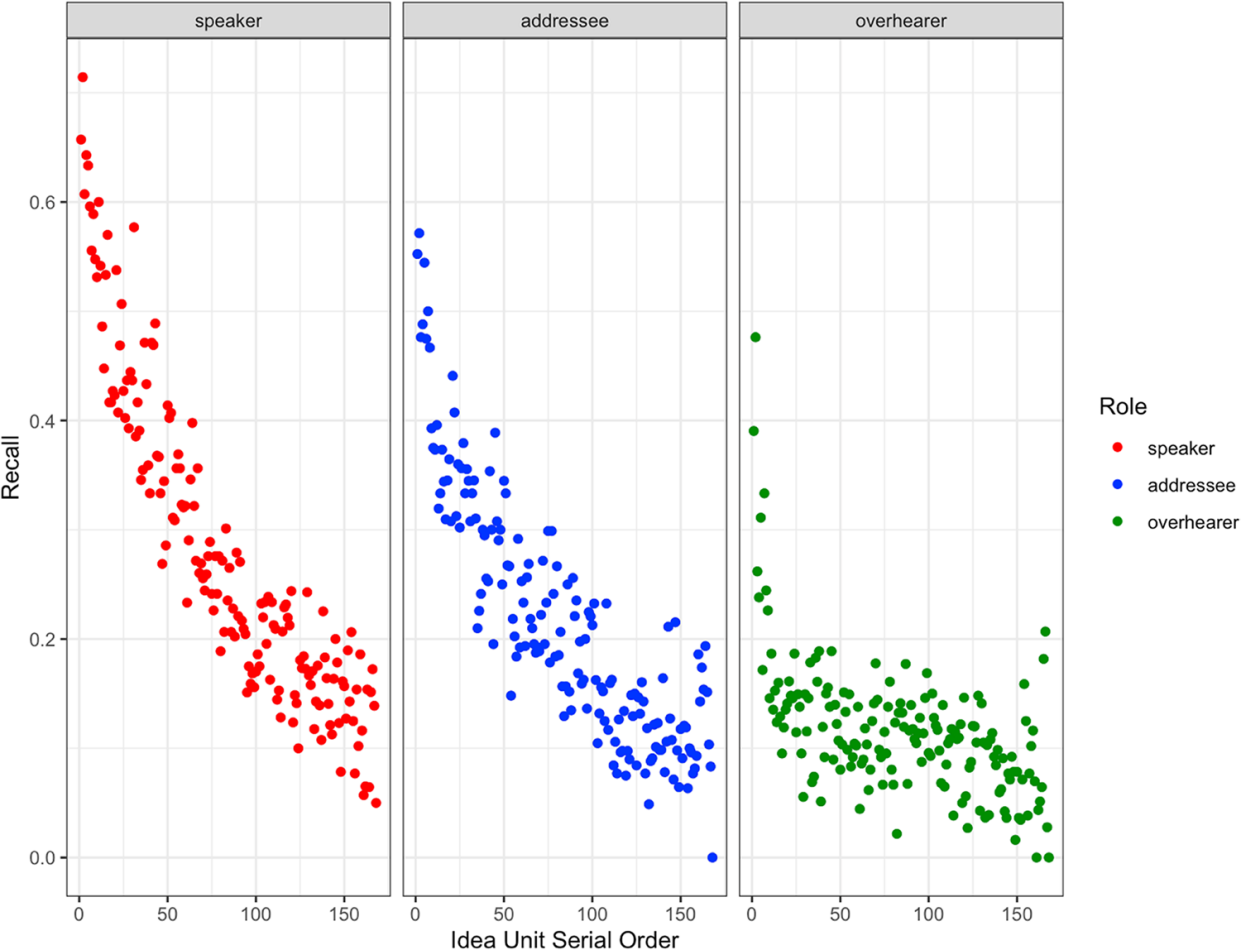
Recall probability as a function of Idea Unit Serial Order (for illustration purposes, the x-axis is re-scaled such that all conversations fall on a 0:200 point scale). Dots indicate by-participant averages for each role and serial order

The model results indicated that, overall, each individual idea unit was more likely to be not recalled than recalled (b = -1.76, *p* < 0.0001). A negative serial order effect indicated that idea units earlier in the conversation were more likely to be recalled than those later in the conversation (b = -1.08, *p* < 0.0001; for illustration, see Fig. 2). Longer idea units were more likely to be recalled than shorter idea units (b = 0.30, *p* < 0.0001; for illustration, see Online Supplemental Material (OSM) Fig. S1). Critically, as predicted, active conversational participants were more likely to recall ideas in the conversation compared to overhearers (b = 1.69, *p* < 0.0001), with active participation increasing the odds of recall by 5.39 over overhearing. We also observed a generation benefit to memory, with greater recall for speakers than addressees (b = 0.44, *p* < 0.0001). Generating an utterance increased the odds of recalling that utterance by 1.56 over being the addressee.

#### Accuracy of recall

**Fig. 3:**
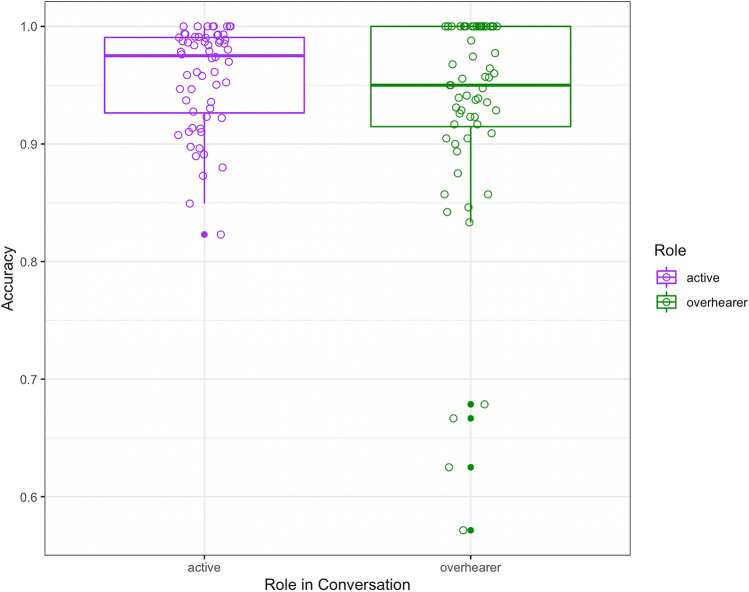
Recall accuracy for the 9473 recall idea units across as a function of participant role (active participant, overhearer). Participant means indicated by circles

The 60 participants in this experiment generated a total of 9,473 recall idea units, of which 95% were accurate reflections of content produced in the conversation (note that this analysis excludes commentaries, which are not easily categorized as accurate or inaccurate). A mixed-effects analysis of recall accuracy included participant role – defined as whether the recaller was an active conversational participant or an overhearer – as a fixed effect. On average, active participants were 95.8% accurate, and overhearers were 92.5% accurate (Fig. 3). Note that whether a given active participant was the speaker or addressee was undefined for inaccurate recall idea units, so that variable is not included in this analysis. Similarly, the control variables of idea unit order and length are undefined for inaccurate idea units and are not included in this analysis. The random effects structure for the model was determined using the buildmer function as before. The final model (Table 4) included random intercepts by participant and group, and a random slope by participant for the Role variable. The intercept was positive (b = 3.28, *p* < 0.0001), indicating that participants were more accurate than not. The effect of participant role was significant (b = 0.44, p = 0.05), corresponding to an increased odds of accurate recall of 1.56 for active participants (speaker and addressee) compared to inactive participants (the overhearer). However, the small effect size and marginal *p*-value suggest that some caution is warranted in interpreting this result.

**Table 4:** Recall accuracy: results of mixed-effects logistic regression analysis for 9,473 recall idea units (60 participants and 20 groups), as a function of participant role (active participant or overhearer)

Fixed effects	Estimate	SE	z-value	*p*-value	OR
(Intercept)	3.279	0.193	16.968	< .0001	26.541
Role (active = .5; overhearer = -.5)	0.443	0.226	1.961	0.050	1.558
Random effects	Variance	SD	Corr		
Participant (intercept)	0.385	0.621			
Role	1.446	1.203	-0.430		
Group (intercept)	0.477	0.691			

#### Source accuracy

**Fig. 4:**
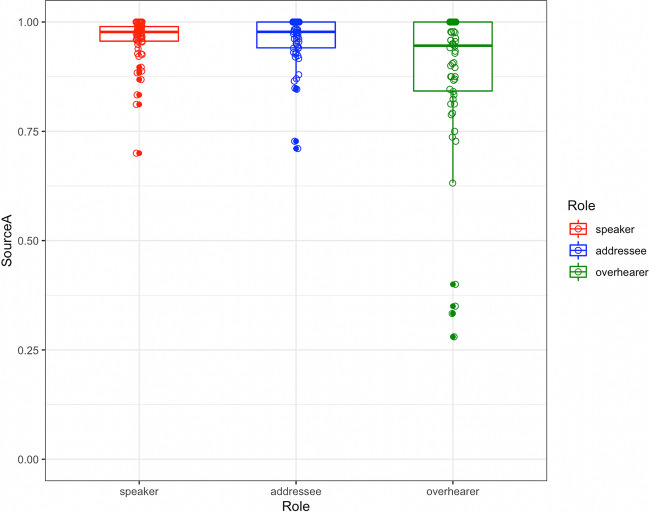
Accuracy of source recall for 8827 recall idea units as a function of participant role (speaker, addressee, overhearer). Participant means indicated by circles

During the recall phase, participants were instructed to recall both the content of the conversation and who said what. Of the 9,012 accurate recall idea units, 8,825 included a source attribution such as “*he mentioned his being a longtime fan of PK Subban*”; “*I noted that he was one of those players that you hate if he’s not on your team*”; or “*Participant A said she also likes musicals.*” The remaining 187 accurate recalls did not have a source attribution and were not included in this analysis (e.g., “*Afterwards, one of the participants asked what the other liked else about Nashville*”). Of the 187 non-attributions, there were a similar number from participants in the role of speaker (64), addressee (57), and overhearer (66). Among the 8,825 idea units included in this analysis, the source identification was highly accurate (Fig. 4), with 96.5% accuracy for speakers; 96.1% for addressees, and 90.0% for overhearers.

A mixed-effects analysis of source accuracy used the same model-fitting procedure as in the analysis of recall completeness. The final model (Table 5) included random intercepts by participant and group, a random slope by participant for the Role variable, and a random slope by Group for the idea unit length variable. The intercept was positive (b = 3.42, *p* < 0.0001), indicating that source identifications were more accurate than not. The first contrast was significant (b = 1.42, *p* < 0.0001), with higher source accuracy for active conversational participants than overhearers; the second contrast for participant role was not significant (b = 0.06, p = 0.63), indicating that accuracy at identifying the source was not significantly different for the person who spoke that idea unit than for the person to whom it was addressed. Lastly, idea unit length (but not serial order) predicted source accuracy, with greater accuracy for longer idea units (b = 0.90, *p* < 0.05).

**Table 5:** Accuracy of source memory for correctly recalled idea units (IUs). Results of mixed-effects logistic regression analysis for 8,825 binary data points (60 participants, and 20 groups), as a function of participant role

Fixed effects	Estimate	SE	z-value	*p*-value	OR
(Intercept)	3.417	0.180	19.005	< .0001	30.470
Role1 (S = .25, A = .25, O = -0.5)	1.417	0.349	4.065	< .0001	4.127
Role2 (S = .5, A = -.5, O = 0)	0.061	0.127	0.479	0.632	1.063
IU order	-0.069	0.083	-0.835	0.404	0.933
IU length	0.901	0.352	2.561	0.010	2.461
Random effects	Variance	SD	Corr		
Participant (intercept)	0.432	0.657			
Role1	4.577	2.139	-0.050		
Group (intercept)	0.381	0.618			
IU length	1.109	1.053	-0.030		

#### Similarity of recall

An exploratory analysis of recall similarity that was not pre-registered quantified whether each individual idea unit was likely to be recalled by none of the partners, all partners, or only by a subset of them (Table 6). This analysis was motivated by recent findings in our lab pointing to a discrepancy between recall completeness and similarity such that when forgetting rates are high, similarity in recall may be high due to common recall failures, even though joint recall is quite low (Brown-Schmidt et al., 2023).

**Table 6:** Similarity of memory. Top row: proportion of conversational idea units (IUs) recalled by neither, one, or both of the active conversational participants. Middle row: proportion of IUs recalled by neither the active participant nor the overhearer, by either the active participant or the overhearer, or by both the active participant and the overhearer; note that this analysis compares the overhearer to each of the active participants *separately*, thus the total number of IUs is doubled. Bottom row: proportion of IUs recalled by all three participants, by one or two of the three participants, or by none of them

	No recall	Partial recall	Joint recall	Total IUs
Active participants	0.59 (7,798 IUs)	0.3 (3,908 IUs)	0.12 (1,533 IUs)	13,239
Active + overhearer	0.67 (17,645 IUs)	0.28 (7,500 IUs)	0.05 (1,333 IUs)	26,478
All three participants	0.54 (7,154 IUs)	0.43 (5,704 IUs)	0.03 (381 IUs)	13,239

Using these similarity metrics, we can calculate a measure of mnemonic similarity that quantifies the similarity of recall by taking into account the idea units that are uniformly recalled or not (indicators of similarity) or recalled by some but not others (Coman et al., 2016). For example, the mnemonic similarity for active conversational partners (MS_A_) is calculated by taking the sum of idea units recalled by both (RR) or not recalled by both (NN), divided by the total; we then calculate the same metrics between each active participant and the overhearer (MS_AO_), and for the group of three participants (MS_G_):

$$\begin{array}{l}{\mathrm{MS}}_{\mathrm A}:\;(\mathrm{RR}+\mathrm{NN})/\;\mathrm{total}\;=\left(1533\;+7798\right)/13239=70.5\%\;\;\;\;\;\;\\{\mathrm{MS}}_{\mathrm{AO}}:\;\left(\mathrm{RR}+\mathrm{NN}\right)/\;\mathrm{total}=\;(1333+17645)/26478\;=71.7\%\;\;\;\\{\mathrm{MS}}_{\mathrm G}:\;\left(\mathrm{RR}+\mathrm{NN}\right)/\;\mathrm{total}\;=(381+7154)\;/13239=\;56.9\%\;\;\;\;\;\;\;\;\;\end{array}$$

These metrics suggest that mnemonic similarity is as similar for pairs of active participants as it is for pairs with one active and one inactive participant. The similarity of the group of three participants is notably lower. While this is almost necessarily the case simply due to chance (concordance among three vectors will nearly always be lower than two of them), it highlights the fact that in group settings, it is unlikely that all members of the group will walk away from that interaction with similar memories of the conversation. What these similarity metrics disguise, however, is that joint recall – the probability that an individual idea unit will be recalled by multiple individuals – is much higher for the two active participants (11.5%) than for pairs of active and inactive participants (5.0%), or to the group of three (2.9%). This finding is likely due to the higher recall rates by active participants reviewed above. Thus, similarity is inflated by the high probability of a recall failure; when we focus on recall success, active engagement enhances similarity among participants.

Inferentially, we conducted a post hoc analysis at the level of groups to compare the memory similarity of active versus non-active conversational participants. This analysis, which was not planned prior to the inspection of the data, used the *addressee’s* memory for each idea unit in each conversation as the dependent measure. The empirical question was whether the *speaker’s* memory for that same idea unit would be a better predictor of listener memory than the *overhearer’s* memory for that same idea unit. If actively engaging in conversation makes memory more similar, speaker memory should be the better predictor. On the other hand, we know that the speaker will benefit from a generation boost to memory, thus if it is simply the action of listening versus speaking that determines memory similarity, then overhearer memory should be a better predictor of addressee memory. This analysis was conducted in a similar way as before. Addressee memory for each idea unit in the conversation was treated as dependent and the inputs to buildmer included idea unit length and order, speaker memory and overhearer memory, as well as all possible random slopes (two idea units were missing in this analysis due to missing data for the person in the overhearer role).

The final model generated by buildmer (Table 7) revealed that Speaker memory was a better predictor of Addressee memory compared to Overhearer memory. When the Speakers remembered an idea unit, the odds that the Addressee would remember it increased by 2.37 (b = 0.86, *p* < 0.0001). In contrast, when the Overhearer remembered an idea unit, the odds that the Addressee would remember it increased by 2.00 (b = 0.69, *p* < 0.0001).

**Table 7:** Similarity of memory. Results of mixed-effects logistic regression analysis for 13,237 binary data points (across 20 groups), as a function of Speaker memory, Overhearer memory, and Idea Unit (IU) order

Fixed effects	Estimate	SE	z-value	*p*-value	OR
(Intercept)	-1.749	0.096	-18.191	< .0001	0.174
Speaker memory	0.862	0.099	8.695	< .0001	2.369
Overhearer memory	0.694	0.061	11.333	< .0001	2.002
IU order	-0.773	0.098	-7.923	< .0001	0.462
Random effects	Variance	SD	Corr		
Group (intercept)	0.165	0.406			
IU order	0.159	0.399	0.560		
Speaker	0.153	0.391	-0.790	-0.100	

## General discussion

This paper examines the completeness and accuracy of conversational recall as a function of conversational role. Inspired by classic theoretical proposals in the study of conversation, we hypothesized that active participation in conversation involves the formation of representations of mutual knowledge (or *common ground*), and that those representations support memory. We predicted that participants who are privy to the entire conversation but experience it in the role of an overhearer are not in a position to participate in the formation of common ground for the conversational exchange (Wilkes-Gibbs & Clark, 1992), and as a result, would have limited memory for the conversation. Prior work has demonstrated that the formation of common ground for collaboratively established image descriptions (as measured by the shortening of those descriptions over time) is positively associated with later memory for the images and labels (McKinley et al., 2017; Nault et al., 2023). The results here demonstrate a related phenomenon at the level of individuals: those who are not part of the collaborative process of forming common ground do not enjoy the memory benefits associated with those cognitive activities.

This result is noteworthy in light of our understanding of the mechanics of speaking in conversation. The fact that the overhearers in our task did not need to pay strict heed to the evolving demands of conversational turn-taking makes it entirely plausible that they would have experienced a benefit in remembering the contents of the conversation. Participating in a conversation requires simultaneous comprehension and planning, and often one must plan a contribution while simultaneously listening to what one’s partner is saying (Sacks et al., 1974; Stivers et al., 2009; Bögels et al., 2015), or monitor an addressee for understanding while speaking (Clark & Krych, 2004). These temporally overlapping demands certainly stand in the way of explicit attempts to remember important details. Although overhearers are not positioned to benefit from the generation and production effects (MacLeod et al., 2010; Slamecka & Graf, 1978), the casual overhearers studied in the present task do not have the simultaneous pressures of planning and monitoring. It is clear from these results that between the competing factors of nonparticipation (which should lessen memory in overhearers) and reduced cognitive load (which should support memory in overhearers), the former is the dominant factor: memory is substantially lessened in individuals who do not participate in a conversation.

Our findings show clear effects of conversational role on memory for conversation. The prior literature examining asymmetries in memory for active conversational participants is somewhat mixed, with a number of studies reporting better memory for speakers compared to addressees (Brown-Schmidt et al., 2023; McKinley, et al., 2017; Miller et al., 1996; Nault et al., 2023; Ross & Sicoly, 1979; Yoon et al., 2016, 2021), and other studies finding no difference or even a *listener* benefit (Benoit & Benoit, 1988; Knutsen & Le Bigot, 2014; Stafford & Daly, 1984; Stafford et al., 1987). Here we find again that conversational participants recall significantly more of what they said themselves compared to what was said to them. Overhearers recalled significantly less conversational content. The locus of this effect may arise from a confluence of factors, including overhearers not actively participating in the formation of common ground, conversational topics that were not of interest to the overhearer, and a lack of engagement with the conversation itself while the overhearer engaged in a secondary task.

We also observed considerable variability in performance (see by-person means in Fig. 1), with the best-performing overhearer recalling 37.74% of the conversation, and the worst-performing overhearer recalling only 0.46%. By contrast, the best-performing conversational participant in any one conversation recalled 44.12% of what was said overall, and the worst performing only 7.37%. The fact that some individuals, when in the role of overhearer, exhibited recall rates comparable to some active conversational participants, indicates that active participation in conversation is not a pre-requisite to remembering conversation. Further, the fact that the *best* performing active conversational participant recalled less than half of what was said after a mere 20-min delay highlights the fact that memory for conversation is often limited at best. These and related findings (Benoit & Benoit, 1988; Brown-Schmidt et al., 2023; Ross & Sicoly, 1979; Stafford & Daly, 1984; Stafford et al., 1987) challenge classic views that point to episodic memory representations of the discourse history as the basis for inferences about what information is and is not in common ground with one’s discourse partner (Clark & Marshall, 1978). Instead, the present findings emphasize the need for future research examining how asymmetries in memory for conversation among conversational partners shape beliefs about shared experiences, in turn influencing future communicative behavior.

The present experiment examined dyadic conversation in which the conversational participants were aware of the presence of a co-present but non-participating overhearer who was simultaneously playing a computerized chess game. In everyday settings, co-present overhearers will frequently be concurrently engaged in another task, such as overhearing a conversation while perusing a magazine at a doctor’s office or while eating lunch at a café. Overhearing may occur in more charged situations as well, such as when overhearing a domestic dispute between one’s neighbors. The particular conversation being overheard and the task being performed likely compete for attention, and the trade-off is a function of the relative importance of the two activities. For example, listening to a recording of a conversation in one’s native language had little impact on performance of a concurrent multiple-object-tracking task, though listening in one’s second language did (Kunar et al., 2018). Listening to a conversation did not impact performance in a task that involved searching for words in a table (though listening to a one-sided conversation, as when overhearing a telephone conversation, did; Marsh et al., 2018). Cataloging the impact of different types of concurrent tasks on overhearer memory for conversational language remains an open area for future research. Likewise, future research may explore different approaches to the task of overhearing, ranging from the casual overhearers examined in the present work, to more intentional overhearing, as in cases where a person intentionally eavesdrops or wiretaps a conversation.

In other situations, the group dynamics may be such that conversational participants have task-related coalitions or other relationships that allow common ground to be formed with non-active members of the group by proxy, in which a coalition member provides feedback indicating that the coalition understood what was said (Eshghi & Healey, 2016). Group dynamics, including whether partners contribute equally to conversation, and whether there are misunderstandings may also be relevant to conversational memorability (Guydish & Fox Tree, 2023). In formal settings such as legal proceedings, wedding vows, and other public declarations, the roles that participating individuals play become even more complex. For example, consider the role of the judge and jury who observe a prosecutor interrogating a witness. In such cases, the prosecutor and witness are speaking to each other, but their utterances are also designed in a way so as to be interpretable to the judge and jury – so-called *institutional witnesses* (Clark & Carlson, 1982). For example, an expert witness may define key terms or answer a technical question using lay terminology in a way that is intended to be interpretable to judge and jury. Institutional and social dynamics in the broader situation may be relevant to an overhearer’s later memory for that experience, along with other factors such as whether note-taking at trial is allowed (see Forsterlee et al., 2005; Sevier, 2011).

It is noteworthy that, in terms of accuracy of what was recalled, active participants were only slightly more accurate than overhearers (95.8% vs. 92.5%), and that both groups were highly accurate. The finding that active participation in conversation confers an advantage in the quantity of information that can be recalled after a ~ 20-min delay, with minimal advantages in the accuracy of what is recalled, is reminiscent of recent evidence that retrieval practice for conversational memory (i.e., having recalled that conversation a week earlier) increases the amount of information that can be accurately recalled from conversation, but does not significantly increase the accuracy of that recall (Brown-Schmidt et al., 2023). One explanation of the present finding is that active participation confers a variety of mnemonic benefits (including self-relevance, generation, heightened attention) that increase the probability of recall, but that general knowledge of the gist of what was discussed is shared by active participants and overhearers alike, and this gist prevents intrusions and other inferential mistakes that might lead to inaccurate recall. Overhearers, like active conversational participants, may have similarly high estimated accuracy thresholds for reporting information, resulting in high accuracy even in the face of lower recall. It is noteworthy that source memory accuracy seemed to pattern differently than accuracy of the content of what was recalled. For accurately recalled idea units, active participants were significantly more accurate in their judgments of source compared to overhearers (96% vs. 90%). It is possible that the inferential processes that support knowledge of the gist of the conversation more strongly supports recall of content than memory for source.

While it was not a focus of the present analysis, overhearers produced a greater proportion of commentary compared to active participants (21% vs. 13%). Overhearers may have opted to produce more general observations at a larger grain size when unable to recall additional conversational details (see Goldsmith et al., 2002, and Hamilton et al., 2023, for related discussion). We also observed variability in recall style, including whether participants used direct or indirect quotes (see Clark & Gerrig, 1990), whether they indicated confidence, e.g., “I *think* that’s where she’s from,” and whether they mentioned broader aspects of the conversational situation, e.g. “I spoke first.” While outside the scope of the present article, exploring the impact of these different recall styles on recall quality may prove fruitful. Similarly, whereas the present work focuses on gist recall of the ideas in conversation, examining verbatim recall of conversation may offer additional insights into the effects of role on recall quality.

When comparing the similarity of memory across participants, we find that, on average, pairs of active participants jointly recalled more idea units than pairs of active and inactive participants (11.5% vs. 5.0%), and that the percent of idea units accurately recalled by all three individuals was only 2.9%. These findings emphasize that, particularly when one person is not actively involved in conversation, very few of the basic ideas in that conversation will be recalled by all of the individuals who were witness to it. These findings provide some context to thinking about what we may expect when comparing witnesses’ memory for conversation. These findings suggest that joint recall of conversation by multiple witnesses is likely to be limited to a small proportion of the total talk that occurred.

More broadly, we note that differences in recall completeness, recall accuracy, and source accuracy between active conversational participants and overhearers may have relevance in applied settings, including the courtroom. The US legal system generally trusts jurors to evaluate and appropriately weigh evidence (including witness testimony). But evidentiary rules, such as the oft-discussed hearsay rules, may prohibit the jury from considering certain types of evidence – particularly evidence thought to be of dubious reliability (Imwinkelried, 1989; Sevier, 2014; Tribe, 1974).

While many evidentiary rules track intuitions about reliability, our findings suggest concerns over the reliability of overheard statements that are not currently reflected in evidentiary rules. Neither hearsay doctrine nor other evidentiary rules distinguish between active participants in a conversation and overhearers. If witness testimony as to an *overheard* out-of-court statement, like CJ’s testimony about Joey Watkins’ alleged confession, is not barred by an evidentiary rule (e.g., if it falls into an applicable exclusion or exception under the hearsay rules), the testimony will be as admissible as if the out-of-court statement were made to or by the witness. Of course, the fact that testimony is admitted does not mean the jury will afford it great weight. Indeed, there is empirical evidence that, in some circumstances, mock jurors understand potential problems with hearsay evidence and appropriately discount such evidence when rendering a verdict (Kovera et al., 1991; Miene et al., 1992; Sevier, 2021). It is possible that jurors may make similar adjustments when considering testimony about overheard statements. A good opposing lawyer would highlight for the jury the fact that the witness merely overheard the statement, with the idea that the jurors would (i) intuitively understand that overhearers are less likely to accurately and completely remember statements and sources than active conversational participants, and (ii) discount the weight they give the witness’s testimony accordingly. But do jurors discount evidence of overheard statements? If so, do they discount such evidence to an appropriate degree? Future research might empirically evaluate what is potentially a complicated metacognitive judgment (Brown-Schmidt et al., 2023; Jaeger et al., 2017). If lay decisionmakers systematically overestimate the value of testimony about overheard utterances, policymakers might consider tailoring evidentiary rules that limit juries’ reliance on overheard statements, at least in some instances.^7^ This may also be an area where expert testimony about attention, perception, and/or memory would be particularly useful to jurors – potentially more useful than judges may recognize (Schmechel et al., 2005).

### Constraints on generality

The present study examined conversational memory in a sample of young, English-speaking adults in a university setting. We focus on the impact of conversational role at the time of conversation on individuals’ subsequent memory for that exchange. We do not examine social processes at the time of recall that may impact remembering in social settings (see Abel & Roediger, 2018; Coman, et al., 2009; Hirst & Echterhoff, 2012). How the present findings may extend to individuals of different ages and in different social and linguistic contexts also remains an open question for future research.

Critically, much more research would be needed to link the effects reported here to courtroom situations like the Joey Watkins case. Such future work might address three noteworthy limitations of our initial study. First, the overheard conversations in our study typically involved relatively mundane topics such as music and television shows. (Thankfully, no study participants confessed to a murder.) While mundane conversations can have legal relevance, it would be important to study overhearers’ recall for more important or contextually surprising statements like confessions. It may be that memory differences between overhearers and conversational participants diminish when the overheard statement is particularly engaging, surprising, inappropriate, or otherwise attention-grabbing (Keenan et al., 1977; Kintsch & Bates, 1977). Second, and relatedly, participants in our study had no reason to believe that they needed to retain overheard information at the time they were overhearing it. In contrast, in some legal cases (like the Watkins case), it may be immediately evident to an overhearer that they should retain the relevant information. For example, Bast (1997) discusses several cases in which a person accidentally hears a noteworthy conversation (e.g., a cordless telephone conversation about drug sales picked up by a radio^8^), and then records or reports the conversation to authorities. An overhearer’s recognition that the statement they heard may need to be reported to law enforcement officials or in a courtroom may lead them to use cognitive strategies that reduce or eliminate recall deficits. Relatedly, a neutral third-party overhearer such as an individual who accidentally overhears a conversation about illegal activity may be perceived by the court or factfinder to offer a more neutral perspective on the event, compared to an active participant in that conversation who may be perceived to have been biased. In such situations, there may be multiple reasons why testimony from an overhearer would be preferred to that of an active participant. Third, participants in our study were engaged in a fairly cognitively demanding task – playing a game of chess – as they overheard a conversation. In the real world, overhearers might be involved in tasks that involve significantly less demand for cognitive resources (e.g., waiting in line at a store) or that involve the need for more or different types of cognitive resources (e.g., playing a visually intensive shooting video game). Future research might vary the overhearers’ activity or measure performance on that activity to examine the effects on overhearer recall. Future research could also probe the extent to which varying what lay decision-makers are told about the overhearers’ activity affects how they value overhearers’ testimony.

## Conclusion

The present work reports the results of a novel experiment examining memory for conversation in group settings. Consistent with prior empirical evidence that forming common ground for an individual element of conversation supports memory for it, as well as theoretical arguments that overhearers are limited in the degree to which they can form common ground for conversation, we find that overhearers recall significantly less content from conversation compared to both speakers and addressees. While overhearers are nearly as accurate as active conversational participants in the content of their recalls, their ability to accurately attribute a given piece of content to an individual is significantly less accurate. The ability to accurately and completely recall a conversation is relevant to daily activities such as keeping up with friendships and being a successful communicator, but can also be relevant in certain business, law enforcement, political, and legal settings where there is a need to uncover the content of a past communicative exchange. Here, we show that active conversational participants – and in particular, speakers – are best positioned to provide a complete recall of a prior conversation, compared to a person who overheard the same exchange.

## Supplementary Information

Below is the link to the electronic supplementary material.

Supplementary file1 (PDF 55 KB)

## Footnotes

1 https://undisclosed-podcast.com/docs/season-2/213/Statement%20of%20Corey%20Jacobs%20-%206-21-00.pdf

2 https://undisclosed-podcast.com/docs/season-2/213/Testimony%20of%20Corey%20Jacobs%20-%20Watkins%20Trial.pdf

3 For more recent developments in this case, *Watkins v. Ballinger*, 308 Ga. 387, 840 S.E.2d 378 (Ga. 2020), see https://www.georgiainnocenceproject.org/active-cases/joey-watkins/

4 https://clinton.presidentiallibraries.us/memcons-telcons

5 https://nsarchive.gwu.edu/document/16116-document-05-memorandum-conversation-between

6 https://www.graphpad.com/quickcalcs/kappa1/

7 In considering such rules, policymakers would need to balance concerns about witness’s memory for overheard statements with other policy concerns that animate evidentiary rules (Sevier, 2021). For example, while a witness may be more likely to *misremember* overheard assertions than conversational participants, they might often be less likely to *fabricate* out-of-court assertions than conversational participants (assuming overhearers are less likely to know speakers than conversational participants).

8 State v. Howard, 679 P.2d 197 (Kan. 1984).

## Declarations

### Open practices statement

This study was pre-registered at: https://osf.io/pjv2c. The deidentified data and analysis scripts are available at: https://osf.io/w98jb/.
