# Supplementary material for: Remembering conversation in group settings
Source: Mem Cognit. 2024 Sep 5;53(4):1037–54. doi: 10.3758/s13421-024-01630-8 (PMC12141120; doi:10.3758/s13421-024-01630-8)
Supplement: Supplementary file 1 — Supplementary file1 (PDF 55 KB) [file 13421_2024_1630_MOESM1_ESM.pdf]

Supplemental Materials

Citation: Brown-Schmidt, S., Jaeger, C. B., Lord, K., & Benjamin, A. S. (in press). Remembering Conversation in Group Settings. *Memory & Cognition.*

Contact: sarah.brown-schmidt@vanderbilt.edu

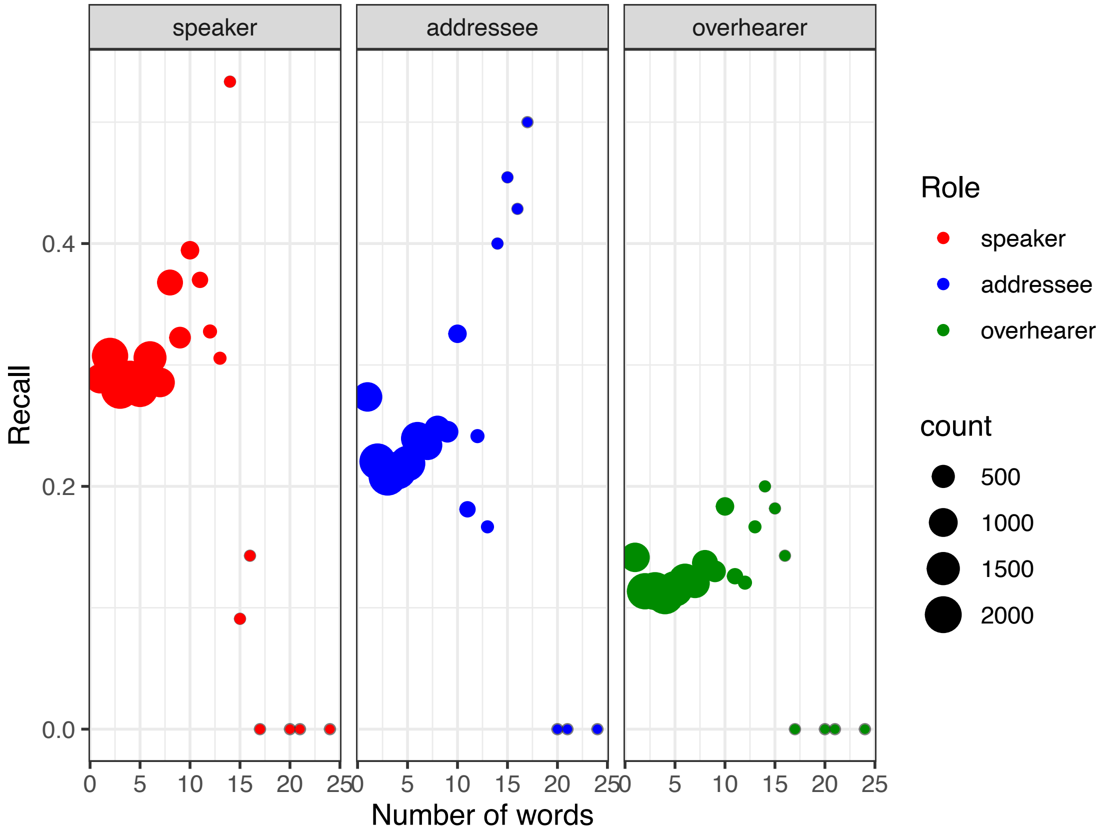

Figure S1. *Recall probability as a function of Idea Unit length (in words)*
